# Potent Anticancer Effect of the Natural Steroidal Saponin Gracillin Is Produced by Inhibiting Glycolysis and Oxidative Phosphorylation-Mediated Bioenergetics

**DOI:** 10.3390/cancers12040913

**Published:** 2020-04-08

**Authors:** Hye-Young Min, Honglan Pei, Seung Yeob Hyun, Hye-Jin Boo, Hyun-Ji Jang, Jaebeom Cho, Ji Hye Kim, Jaekyoung Son, Ho-Young Lee

**Affiliations:** 1 Creative Research Initiative Center for Concurrent Control of Emphysema and Lung Cancer, College of Pharmacy, Seoul National University, Seoul 08826, Korea; 2 Department of Molecular Medicine and Biopharmaceutical Sciences, Graduate School of Convergence Science and Technology and College of Pharmacy, Seoul National University, Seoul 08826, Korea; 3 College of Pharmacy and Research Institute of Pharmaceutical Sciences, Seoul National University, Seoul 08826, Korea; 4 Department of Biomedical Sciences, Asan Medical Center, AMIST, University of Ulsan College of Medicine, Seoul 05505, Korea

**Keywords:** gracillin, breast cancer, lung cancer, glycolysis, oxidative phosphorylation, phosphoglycerate kinase 1

## Abstract

Metabolic rewiring to utilize aerobic glycolysis is a hallmark of cancer. However, recent findings suggest the role of mitochondria in energy generation in cancer cells and the metabolic switch to oxidative phosphorylation (OXPHOS) in response to the blockade of glycolysis. We previously demonstrated that the antitumor effect of gracillin occurs through the inhibition of mitochondrial complex II-mediated energy production. Here, we investigated the potential of gracillin as an anticancer agent targeting both glycolysis and OXPHOS in breast and lung cancer cells. Along with the reduction in adenosine triphosphate (ATP) production, gracillin markedly suppresses the production of several glycolysis-associated metabolites. A docking analysis and enzyme assay suggested phosphoglycerate kinase 1 (PGK1) is a potential target for the antiglycolytic effect of gracillin. Gracillin reduced the viability and colony formation ability of breast cancer cells by inducing apoptosis. Gracillin displayed efficacious antitumor effects in mice bearing breast cancer cell line or breast cancer patient-derived tumor xenografts with no overt changes in body weight. An analysis of publicly available datasets further suggested that PGK1 expression is associated with metastasis status and poor prognosis in patients with breast cancer. These results suggest that gracillin is a natural anticancer agent that inhibits both glycolysis and mitochondria-mediated bioenergetics.

## 1. Introduction

Despite many efforts to develop efficacious anticancer therapeutics, cancer is one of the leading causes of human death worldwide [1]. The genetic and molecular heterogeneity of cancer cells mediates resistance to anticancer therapies, eventually leading to recurrence and poor prognosis [2]. Because the phenotypes of cancer cells involve the convergence of such cellular changes, understanding the biology of cancer evolution is thus important to reduce the social and economic burden caused by cancer [3,4]. Cancer cells rewire metabolic pathways and supply themselves with energy and nutrients to support their aggressive phenotypes [5,6,7,8,9]; typical metabolic reprogramming in cancer cells, such as aerobic glycolysis [10], has been suggested to be a hallmark of cancer [11]. Thus, targeting dysregulated metabolism in cancer cells would be beneficial for efficacious anticancer treatment. Several preclinical and clinical studies have been conducted to evaluate the efficacy of anticancer agents targeting cancer metabolism [12,13,14]. For example, the mitochondria complex I inhibitor metformin is effective as an adjuvant anticancer therapy [15]. However, the clinical benefits of metabolism modulators in anticancer therapy need to be evaluated in further studies. In addition, the discovery of novel agents targeting cancer metabolism is also required to improve efficacy and reduce side effects and toxicity.

Cancer cells utilize various bioenergetic pathways according to their genetic and molecular profiling and surrounding microenvironment [16,17,18]. In general, cancer cells tend to rely on aerobic glycolysis, a phenomenon involving the conversion of glucose to lactate under aerobic conditions, to generate energy and utilize the intermediates of glycolysis as building blocks for cellular proliferation [19]. The dysregulation of mitochondrial metabolism is a feature of metabolic rewiring in cancer cells [10,14]. However, aerobic glycolysis is not enough to fully replace oxidative phosphorylation (OXPHOS) for energy production [16,20,21,22]. The blockade of glycolysis by glucose deprivation or the inhibition of glycolytic enzymes has been found to cause the metabolic switch from aerobic glycolysis to OXPHOS [23,24]. Moreover, mitochondrial respiration has been found to be upregulated in cancer cells [25]. Therefore, the inhibition of both aerobic glycolysis and OXPHOS may produce more potent anticancer effects than the inhibition of one of these metabolic pathways alone. Indeed, the dual targeting of both glycolysis and OXPHOS displays efficient antitumor effects, indicating that this combinatorial blockade can be an efficacious anticancer strategy [26,27].

Natural products are considered an important source for the development of anticancer therapeutic agents [28]. In this regard, the present study examined the inhibitory effect of gracillin, a natural product-derived steroidal saponin, on aerobic glycolysis in human breast and lung cancer cells, two major cancer types that are leading causes of cancer-related death worldwide [29]. Previous studies demonstrated that gracillin has a broad-spectrum anticancer effect in a panel of human cancer cells by inducing apoptosis [30,31,32]. We recently demonstrated the inhibitory effect of gracillin on mitochondrial complex II-mediated energy production in non-small cell lung cancer (NSCLC) [32], suggesting the potential of gracillin as a mitochondria-targeting anticancer agent. However, the effect of gracillin on glycolysis has not been explored. Here, we show the capacity of gracillin to inhibit both glycolysis and OXPHOS-mediated bioenergetics, as demonstrated by the elevation of mitochondrial reactive oxygen species (ROS) production and inhibition of cellular adenosine triphosphate (ATP) production, basal oxygen consumption rate, basal extracellular acidification rate, lactate production, and glycolysis-associated metabolite production. Consistent with our previous findings [32], gracillin displayed a significant inhibitory effect on the growth of breast cancer cell lines and patient-derived tumor xenografts without detectable toxicity. Molecular docking analysis using SwissDock suggested that phosphoglycerate kinase 1 (PGK1) is a potential target by which gracillin suppresses glycolysis. The potential of PGK1 as a target for the treatment of breast cancer was found by analyzing publicly available datasets. We found that the PGK1 expression is associated with the metastasis status and the poor prognosis of patients with breast cancer. These results suggest that gracillin is a natural anticancer agent that inhibits both glycolysis and mitochondria-mediated bioenergetics.

## 2. Results

### 2.1. Gracillin Inhibits Cellular Bioenergetics by Disrupting Both Glycolysis and Mitochondrial Function

**Figure 1:**
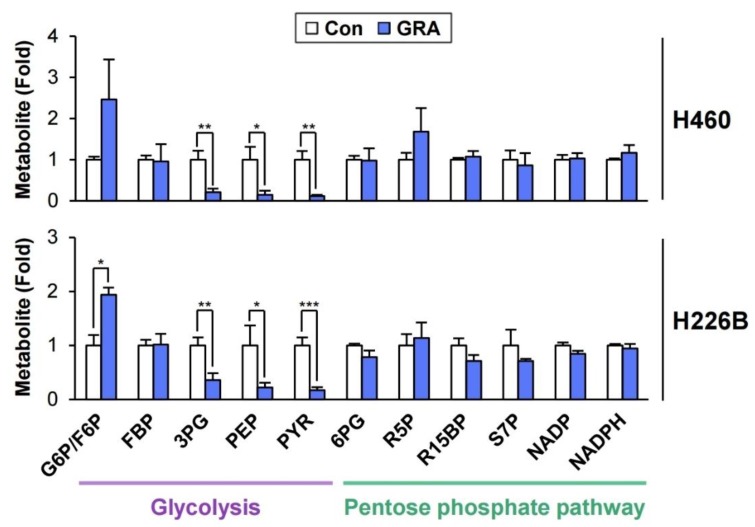
Modulation of glycolysis-associated metabolites by treatment with gracillin. H460 and H226B cells were treated with gracillin for 8 h. Cellular metabolic profiles associated with glycolysis and the pentose phosphate pathway were determined by LC/MS analysis. The bars represent the mean ± SD; * *p* < 0.05, ** *p* < 0.01, and *** *p* < 0.001, as determined by a two-tailed Student’s *t*-test by comparison with the vehicle-treated control group. Con: control; GRA: gracillin; G6P/F6P: glucose-6-phosphate/fructose-6-phosphate; FBP: fructose-1,6-bisphosphate; 3PG: 3-phosphoglycerate; PEP: phosphoenolpyruvate; PYR: pyruvate; 6PG: 6-phosphogluconate; R5P: ribulose-5-phosphate; R15BP: ribose-1,5-bisphosphate; S7P: sedoheptulose-7-phosphate; NADP: nicotinamide adenine dinucleotide phosphate; NADPH: reduced nicotinamide adenine dinucleotide phosphate.

We recently demonstrated the inhibitory effect of gracillin on mitochondria-mediated energy production in NSCLC cells [32]. In the current study, we assessed the effect of gracillin on glycolysis in NSCLC cells. Based on the benefit of targeting both glycolysis and OXPHOS for anticancer therapy [26,27] and a previous study demonstrating the inhibitory effect of the anticancer agent lonidamine on both mitochondrial complex II activity and glycolysis [33], we investigated the effect of gracillin on glycolysis. The LC/MS-based analysis of metabolites in gracillin-treated cells revealed the accumulation of glucose-6-phosphate/fructose-6-phosphate (G6P/F6P), which might be mediated by either activation of hexokinase or inhibition of phosphofructokinase-1, and the marked downregulation of glycolysis-associated metabolites, such as 3-phosphoglycerate, phosphoenolpyruvate, pyruvate, and lactate, in gracillin-treated lung cancer cells compared with vehicle-treated control cells (Figure 1). Metabolites associated with the pentose phosphate pathway were not altered by treatment with gracillin. These results suggest that glycolysis is disrupted in NSCLC cells by gracillin treatment.

**Figure 2:**
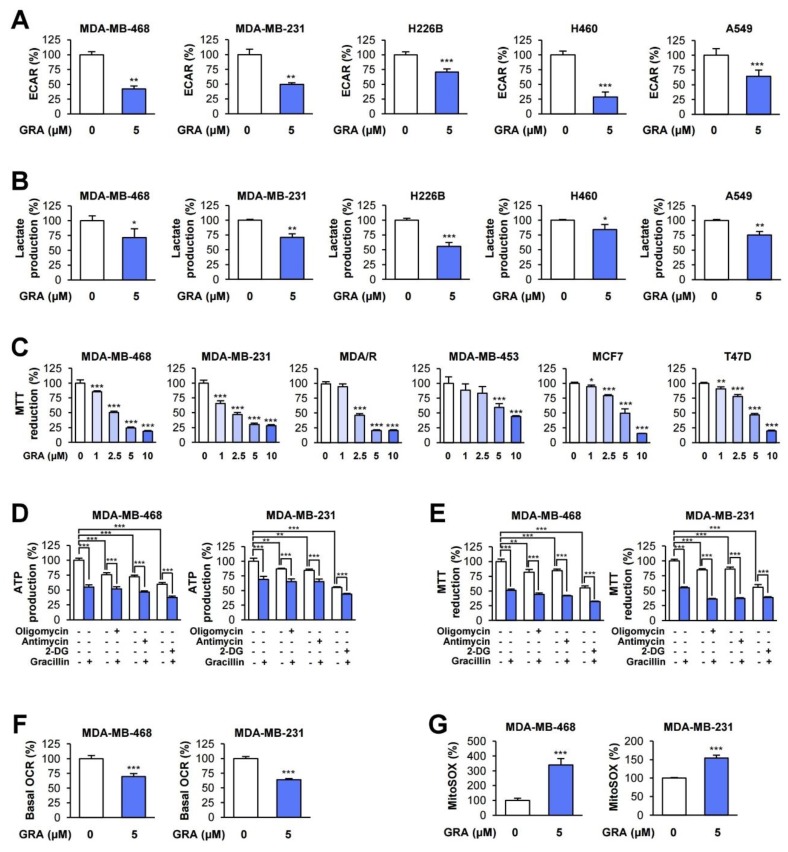
Inhibitory effect of gracillin on glycolysis and OXPHOS. (**A**–**F**) The effect of gracillin on basal extracellular acidification rate (ECAR) (**A**) and oxygen consumption rate (OCR) (**F**) was determined by using a Seahorse XF analyzer. (**B**) The inhibitory effect of gracillin on lactate production in lung and breast cancer cells was determined using a commercially available kit. (**C**) The 3-(4,5-dimethylthiazol-2-yl)-2,5-diphenyltetrazolium bromide (MTT) assay was performed to determine the effect of gracillin on dehydrogenase activity in mitochondria in breast cancer cells. Cells were treated with increasing concentrations of gracillin for three days. (**D**,**E**) Triple-negative breast cancer (TNBC) cells were treated with oligomycin (5 μM), antimycin (10 μM), or 2-deoxy-D-glucose (2-DG; 25 mM) alone or in combination with gracillin (5 μM) for 6 h. The levels of adenosine triphosphate (ATP) production (**D**) and the MTT reduction (**E**) were determined as described in Materials and Methods. (**G**) The upregulation of mitochondrial reactive oxygen species (ROS) by treatment with gracillin was determined using the fluorescent probe MitoSOX as described in Materials and Methods. The bars represent the mean ± SD; * *p* < 0.05, ** *p* < 0.01, and *** *p* < 0.001, as determined by a two-tailed Student’s *t*-test by comparison with the indicated group (**D**,**E**) or vehicle-treated control group (**A**–**C**,**F**,**G**). Con: control; GRA: gracillin.

Previous studies have demonstrated the crucial role glycolysis plays in the growth, survival, and metastasis of various human cancers, including breast cancer [1,34,35]. In particular, glycolysis plays an important role in the survival and metastasis of triple-negative breast cancer (TNBC) [34,36,37,38], an aggressive type of breast cancer with a poor prognosis [39], as well as NSCLC [40,41]. Therefore, we investigated the inhibitory effects of gracillin on glycolysis in several TNBC (MDA-MB-468 and MDA-MB-231) and NSCLC, including squamous (H226B), large (H460), and adenocarcinoma (A549) cancer cell lines. Treatment with gracillin significantly suppressed the basal extracellular acidification rate (ECAR) (Figure 2A) and lactate production in both NSCLC and TNBC cells (Figure 2B), indicating the antiglycolytic effect of gracillin in TNBC and NSCLC cells.

We have previously shown the inhibitory effect of gracillin on mitochondrial complex II-mediated energy production in NSCLC [32]. Hence, we investigated whether gracillin disrupts OXPHOS-mediated cellular energy production in TNBC cells. The 3-(4,5-dimethylthiazol-2-yl)-2,5-diphenyltetrazolium bromide (MTT) assay revealed that treatment with gracillin for three days dose-dependently suppressed formazan formation as a result of the MTT reduction by dehydrogenase activity [42] in mitochondria in MDA-MB-468, MDA-MB-231, and MDA-MB-231 cells carrying acquired resistance to paclitaxel (MDA/R) (Figure 2C). Similar results were obtained with other types of breast cancer cells, including human epidermal growth factor receptor 2 (HER2)-positive (MDA-MB-453) and luminal A type (MCF7 and T47D) breast cancer cells [43] (Figure 2C).

We next analyzed the contribution gracillin treatment on cytosolic and mitochondrial compartments to cellular ATP synthesis. To this end, MDA-MB-231 and MDA-MB-453 breast cancer cells were treated with gracillin either single or in combination with inhibitors of OXPHOS-mediated ATP synthesis (oligomycin or antimycin) or with glycolysis-mediated ATP synthesis [2-deoxy-D-glucose (2-DG)]. Blockade of OXPHOS-mediated ATP synthesis moderately decreased the ATP production, and 80% cellular ATP remained (Figure 2D). In contrast, blockade of OXPHOS-mediated ATP synthesis led to a 40% inhibition of cellular ATP production. These findings suggest that MDA-MB-231 and MDA-MB-453 cells preferentially utilize glycolysis for cellular ATP generation which was consistent with the finding in a previous report [44] In the same experimental conditions, the level of the MTT reduction was well correlated with that of the ATP production (Figure 2E). When we determined the effects of gracillin on glycolysis and OXPHOS-mediated ATP generation, combined treatment of gracillin with oligomycin or antimycin or with 2-DG exhibited greater inhibitory effects on ATP production and MTT reduction than did single treatment. These findings confirmed the inhibitory effect of gracillin on both glycolysis and OXPHOS. The basal oxygen consumption rate (OCR), as an indicator of mitochondrial respiration [45], was also substantially downregulated in gracillin-treated TNBC cells (Figure 2F). Because dysregulated mitochondrial function leads to reactive oxygen species (ROS) generation [46], we also examined whether gracillin generates mitochondrial ROS as an indicator of mitochondrial dysfunction using fluorescence probes to specifically detect mitochondrial superoxide (MitoSOX) [47]. As expected, gracillin reduced the mitochondrial membrane potential and increased mitochondrial ROS (Figure 2G), indicating disrupted mitochondrial function in gracillin-treated breast cancer cells. Collectively, these findings indicate the capacity of gracillin to inhibit both glycolysis and OXPHOS-mediated bioenergetics in TNBC and NSCLC cells.

### 2.2. Gracillin Inhibits the Viability of Human Breast Cancer Cells by Inducing Apoptosis

**Figure 3:**
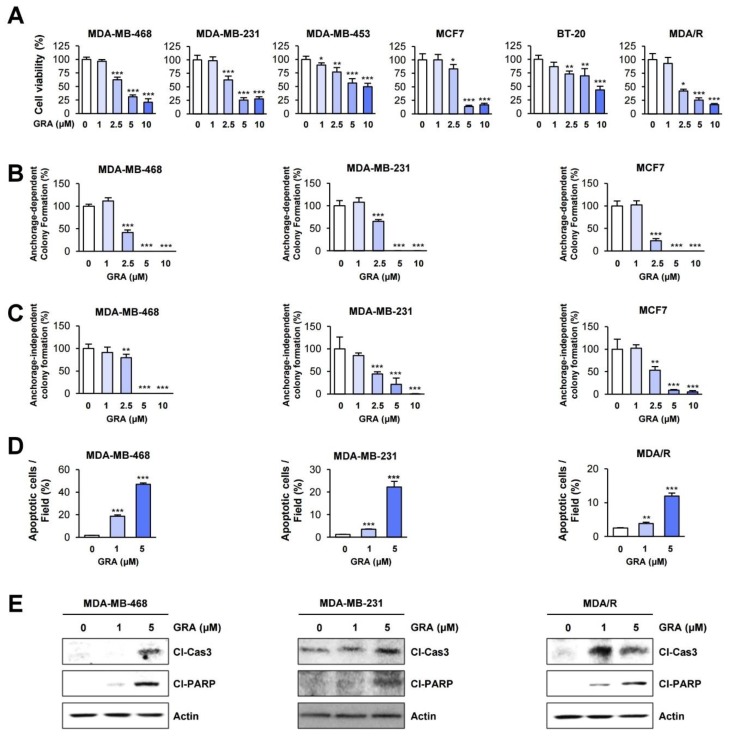
Gracillin exerts inhibitory effects on the viability and colony formation of breast cancer cells by inducing apoptosis. (**A**) The effects of gracillin on the viability of breast cancer cells were determined by crystal violet assay. (**B**,**C**) The effects of gracillin on the colony formation of breast cancer cells were determined by anchorage-dependent colony formation assay (B) and soft agar colony formation assay (**C**). (**D**,**E**) The proapoptotic effects of gracillin were determined by the analysis of Hoechst 33342-stained cells (**D**) and Western blot (**E**). The bars represent the mean ± SD; * *p* < 0.05, ** *p* < 0.01, and *** *p* < 0.001, as determined by a two-tailed Student’s *t*-test by comparison with the vehicle-treated control group. Con: control; GRA: gracillin; Cl-Cas 3: cleaved caspase-3; Cl-PARP: cleaved poly (ADP-ribose) polymerase.

Based on the broad-spectrum antitumor effect of gracillin in vitro and in vivo [30,32], we examined the effect of gracillin on the viability and colony formation of several human breast cancer cells. Crystal violet and colony formation assays revealed that gracillin significantly reduced cell viability (Figure 3A) and colony formation under both anchorage-dependent (Figure 3B) and anchorage-independent (Figure 3C) culture conditions in a dose-dependent manner. Notably, gracillin showed significant cytotoxicity in TNBC cell lines (MDA-MB-231, MDA-MB-468, and BT-20) and those carrying acquired resistance to paclitaxel (MDA/R) (Figure 3A–C), indicating that gracillin may suppress the signaling responsible for aggressive phenotypes in cancer cells. 

We next investigated the mechanism by which gracillin treatment reduces cell viability. Hoechst 33342 staining showed that treatment with gracillin significantly increased the number of cells with condensed chromatin, a feature of cells undergoing apoptosis [48] (Figure 3D). Gracillin also induced a dose-dependent increase in the cleavage of poly (ADP-ribose) polymerase (PARP) and caspase-3 (Figure 3E and Figure S1). These results indicate that gracillin displays potent cytotoxic effects by inducing apoptosis in breast cancer cells.

### 2.3. Gracillin Suppresses Tumor Growth with Minimal Toxicity In Vivo

**Figure 4:**
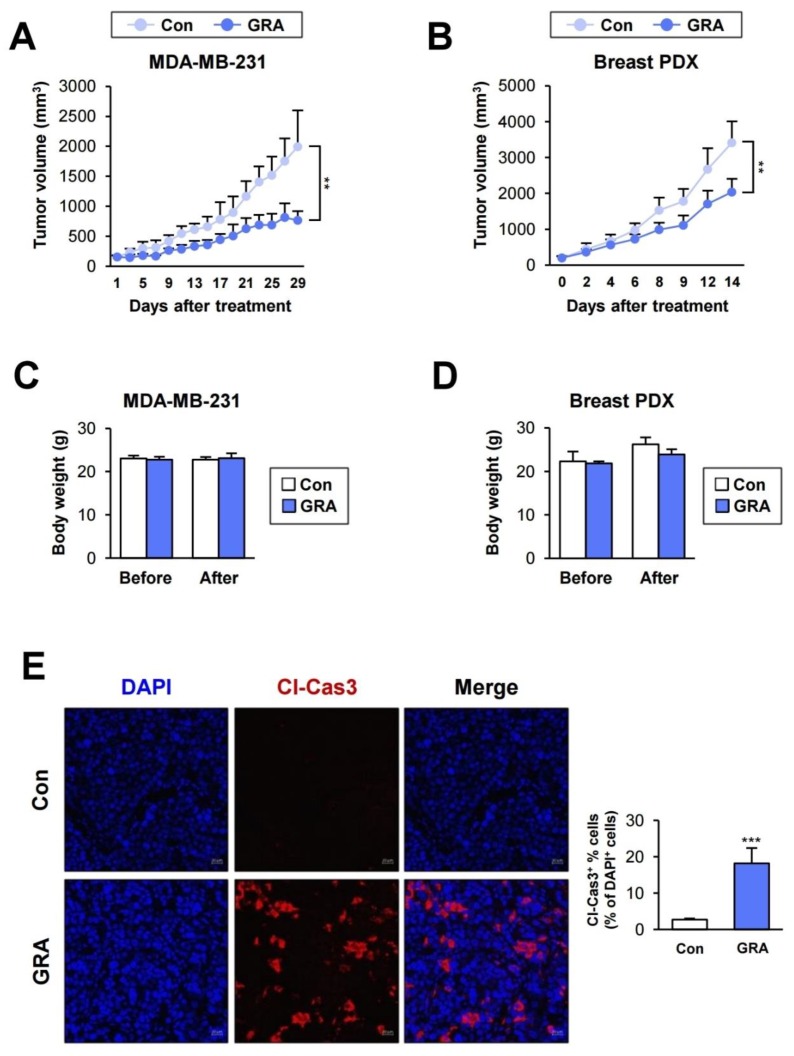
Antitumor effect of gracillin in vivo. (**A**,**B**) Antitumor effects of gracillin in tumor xenograft models established by inoculating MDA-MB-231 cells (**A**) and breast patient-derived tumor tissues (**B**) into mice. (**C**,**D**) Body weight changes in vehicle- or gracillin-treated mice. (**E**) Immunofluorescence analyses of cleaved caspase-3 (Cl-Cas3) expression in patient-derived xenograft (PDX) tumors from vehicle- and gracillin-treated mice. Scale bar: 20 μm. The bars represent the mean ± SD; ** *p* < 0.01 and *** *p* < 0.001, as determined by a two-tailed Student’s *t*-test by comparison with the vehicle-treated control group. Con: control; GRA: gracillin.

We examined the antitumor effect of gracillin in xenograft models using breast cancer cells (MDA-MB-231) (Figure 4A) and patient-derived tumors (Figure 4B). Gracillin significantly inhibited the growth of xenograft tumors. Importantly, the body weights of vehicle- and gracillin-treated mice revealed negligible differences (Figure 4C,D). Immunofluorescence analysis revealed an increase in cleaved caspase-3 (Cl-Cas3) expression in gracillin-treated breast patient-derived xenograft (PDX) tumors (Figure 4E), indicating a proapoptotic effect of gracillin in breast cancer cells in vivo. These results suggest that gracillin has an antitumor effect in a model of human breast cancer and has no detectable toxicity.

### 2.4. PGK1 Is a Potential Target for the Antiglycolytic Effect of Gracillin

**Figure 5:**
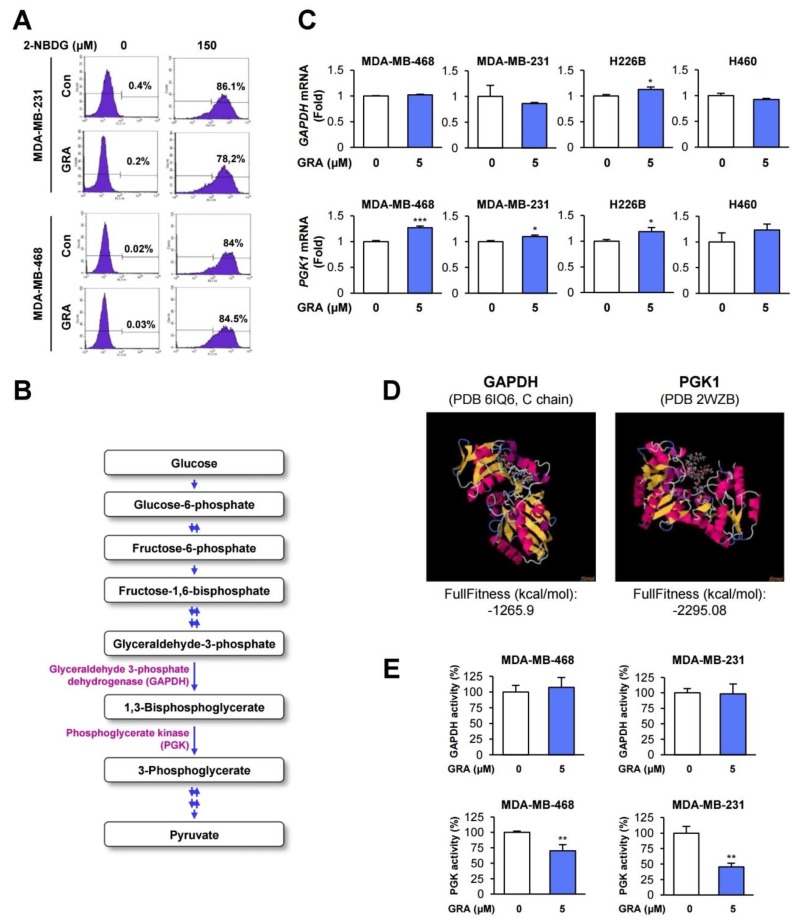
Phosphoglycerate kinase 1 (PGK1) is a potential cellular target for the antiglycolytic effect of gracillin. (**A**) The effect of gracillin on glucose uptake was determined by flow cytometric analysis of 2-deoxy-D-glucose (2-NBDG)-treated cells in the presence or absence of gracillin. (**B**) Schematic diagram illustrating the glycolysis pathway. (**C**) The effect of gracillin on the mRNA expression of *GAPDH* and *PGK1* was determined by real-time PCR. (**D**) Docking analysis of the interaction between gracillin and three glycolysis-associated enzymes (GAPDH and PGK1) using SwissDock. (**E**) The inhibitory effect of gracillin on PGK1 activity with minimal modulation of GAPDH activity was determined as described in Materials and Methods. The bars represent the mean ± SD; * *p* < 0.05, ** *p* < 0.01, and *** *p* < 0.001, as determined by a two-tailed Student’s *t*-test by comparison with the vehicle-treated control group. Con: control; GRA: gracillin.

We investigated the mechanism underlying the inhibitory effect of gracillin on glycolysis. We first determined the effect of gracillin on glucose uptake by using a fluorescent indicator of glucose uptake, 2-deoxy-D-glucose (2-NBDG) [49]. However, flow cytometric analysis revealed that the cellular accumulation of 2-NBDG in the two TNBC cell lines was minimally changed by treatment with gracillin (Figure 5A). Therefore, the antiglycolytic effect of gracillin is not due to the disruption of glucose uptake. 

In the LC/MS analysis of metabolites, we observed the marked downregulation of 3-phosphoglycerate, phosphoenolpyruvate, and pyruvate production in gracillin-treated cells (Figure 1). The decreases in the levels of phosphoenolpyruvate and pyruvate in gracillin-treated cells might be a result of the inhibition of 3-phosphoglycerate production by gracillin treatment. Therefore, the enzymes responsible for producing 3-phosphoglycerate, such as glyceraldehyde 3-phosphate dehydrogenase (GAPDH) and phosphoglycerate kinase (PGK), might be dysregulated by gracillin treatment (Figure 1 and Figure 5B). We determined whether gracillin modulates the mRNA expression of these two glycolysis-associated enzymes (GAPDH and PGK). However, the mRNA expression of these enzymes was not markedly altered by treatment with gracillin (Figure 5C). Since the interaction with small molecules can positively or negatively modulate enzymatic activity [50,51,52], we primarily screened the interaction of gracillin with these two glycolysis-associated enzymes (GAPDH and PGK) by molecular docking analysis using SwissDock [53]. The docking analysis using the crystal structure of GAPDH [the Protein Data Bank (PDB) ID 6IQ6 [54]) and PGK1 (PDB ID 2WZB [55]) revealed that gracillin could interact with these two enzymes, and the interaction of gracillin with PGK1 appears to generate the most stable complex, as shown by the lower full fitness score than that of GAPDH (Figure 5D). Moreover, the enzymatic activity of PGK was significantly inhibited by treatment with gracillin, whereas gracillin minimally affected the activity of GAPDH (Figure 5E). Although additional investigation is required, these results suggest the potential of PGK1 as a target for the antiglycolytic effect of gracillin.

### 2.5. PGK1 Is Associated with Poor Prognosis of Patients with Breast Cancer

**Figure 6:**
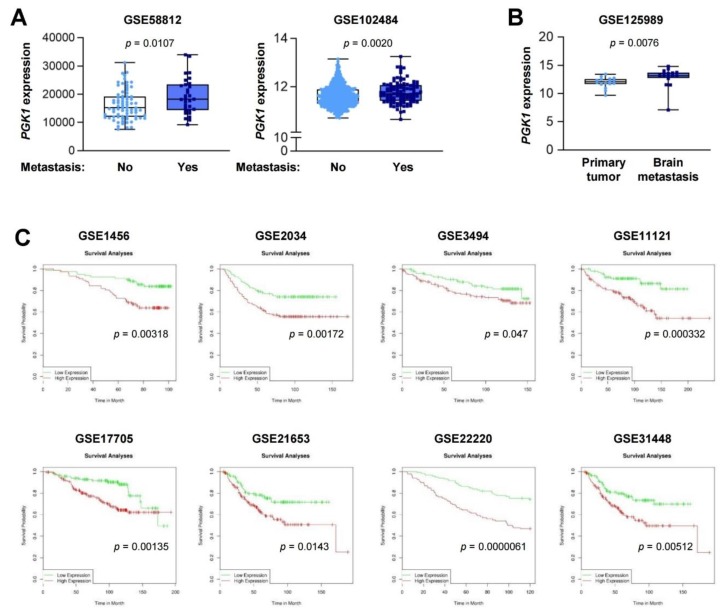
The association of *PGK1* expression with metastasis status and prognosis of patients with breast cancer. (**A**) The association of *PGK1* expression with the metastasis status of breast cancer patients was determined by the analysis of GSE58812 and GSE102484 datasets. The probe of 200738_s_at was used to obtain gene expression values in each dataset. The bars represent the mean ± SD; Statistical significance was determined by a two-tailed Mann–Whitney test. (**B**) Elevated *PGK1* expression levels in brain metastases compared with those in primary tumors was determined by the analysis of a GSE125989 dataset. The probe of 200738_s_at was used to obtain gene expression values in this dataset. The bars represent the mean ± SD; Statistical significance was determined by a Wilkoxon matched-pairs signed-rank test. (**C**) Kaplan–Meier survival curve showing poor overall survival (OS) in the *PGK1*-high population of patients with breast cancer. The survival analysis was performed using DRUGSURV.

Finally, we determined the potential clinical impact of PGK expression on the metastatic status and prognosis of patients with breast cancer by analyzing a public database available in the Gene Expression Omnibus (GEO). We found that tumors derived from breast cancer patients with metastases had higher *PGK1* expression levels than those without metastases (Figure 6A). Analysis of the matched tumor samples from primary tumors and brain metastases also showed higher *PGK1* expression levels in brain metastases than those in primary tumors (Figure 6B). Moreover, patients with breast cancer with high *PGK1* expression displayed a significantly shorter overall survival (OS) duration than those with low *PGK1* expression levels (Figure 6C). These results suggest the potential of PGK1 as a prognostic marker in patients with breast cancer and a therapeutic target for the treatment of breast cancer.

## 3. Discussion

In the present study, we demonstrate the cytotoxic and antitumor effects of gracillin in human breast cancer cells and the potential mechanism of action. We show that, consistent with our recent study [32], gracillin markedly reduces the viability of human breast cancer cells by inducing apoptosis and mitochondrial dysfunction. In addition, gracillin inhibits glycolysis in both breast and lung cancer cells, as evidenced by the modulation of several glycolysis-associated metabolites and significant downregulation of lactate production without affecting glucose uptake. PGK1 is suggested as a potential target for the antiglycolytic effect of gracillin. Furthermore, gracillin displays potent antitumor effects in cell line- and patient-derived tumor xenograft models without overt toxicity. These overall results suggest that gracillin is a novel anticancer agent targeting both mitochondria and glycolysis-mediated bioenergetics in cancer cells. 

Metabolic reprogramming, such as the use of aerobic glycolysis for energy production, has been regarded as one of the hallmarks of cancer [56]. However, recent studies have suggested the role of mitochondria in energy production in cancer cells [16,21,22], metabolic switching under environmental and oncogenic control [16], and the cooperative roles of both aerobic glycolysis and OXPHOS in cancer progression [16], suggesting the importance of targeting both aerobic glycolysis and OXPHOS for anticancer treatment. In support of this hypothesis, combined treatment with glycolysis and OXPHOS inhibitors has been shown to potentiate the antitumor effects [26,27]. Since the combined treatment may cause adverse effects, it is beneficial to use an agent that inhibits both glycolysis and OXPHOS with limited toxicity. In this regard, this study might be important for the development of anticancer agents targeting both glycolysis and OXPHOS-mediated bioenergetics in cancer cells.

First, the present study suggests that, in addition to being a potential mitochondria complex II-targeting anticancer agent [32], gracillin may be a metabolism-targeting anticancer agent that acts by suppressing both glycolysis and OXPHOS-mediated energy production in breast and lung cancer cells. Cancer cells are able to modulate metabolic pathways for energy production and building block generation according to genetic and environmental changes [16]. Supporting this notion, metabolic heterogeneity has been demonstrated in human lung and breast cancer [57,58,59], which might contribute to the aggressive features of these cancer types causing poor prognosis in cancer patients. Moreover, alterations in cancer metabolism can mediate resistance to anticancer therapies in cancer cells [60]. Because aerobic glycolysis and OXPHOS are the main metabolic pathways in cancer cells, it is anticipated that simultaneously targeting both pathways would enhance the antitumor activity. In this regard, gracillin has the potential to be utilized as a potent antitumor agent as a single therapy or in combination with currently available antitumor therapies. The antitumor effectiveness of gracillin in combination with clinically available anticancer agents needs to be evaluated in additional studies.

Through molecular docking analyses and enzymatic activity assays, we suggest that PGK1 is a potential cellular target for the antiglycolytic effect of gracillin. PGK1 is the first ATP-generating enzyme in the glycolytic pathway, which catalyzes the transfer of phosphate from 1,3-diphosphoglycerate to ADP, resulting in the generation of 3-phosphoglycerate and ATP [61,62]. In breast cancer, the expression of PGK1 has been shown to be elevated in malignant tissues compared with corresponding normal tissues and associated with poor clinical outcomes and chemoresistance [63,64,65]. Consistent with these findings, by analyzing publicly available datasets, we also suggest that PGK1 is a prognostic marker for patients with breast cancer. These findings collectively indicate that PGK1 can be considered a target for anticancer therapy. Although several PGK1 inhibitors have been proposed in previous studies [65], specific inhibitors of PGK1 have been poorly defined. Although additional investigation is required, the present study results suggest the possibility that, in addition to being a mitochondrial complex II inhibitor, gracillin can be considered an anticancer PGK1 inhibitor. Considering the requirement of glycolysis for energy production in normal cells, a potential problem of targeting PGK1 might be the nonselective suppression of normal cell viability. Since gracillin displays no obvious toxicity in normal cells derived from various organs and in mice [32], it is possible that gracillin may suppress specifically increased forms of PGK1, such as those modified posttranslationally or mutated, in cancer cells [65,66,67]. In addition, a recent study demonstrated the role of mitochondrial PGK1 in coordinating glycolysis and the TCA cycle in cancer cells [62]. Considering the inhibitory effects of gracillin on both glycolysis and OXPHOS-mediated bioenergetics, mitochondrial PGK1 might be also a potential target for the antiglycolytic effect of gracillin. Together with this aspect, the precise mode of action of gracillin on the inhibition of PGK1 needs to be investigated in further studies.

Along with our previous report, this study also provides a novel metabolism-targeting antitumor agent with clinical applicability and without overt toxicity. In addition to showing antitumor effects on xenografts derived from a human breast cancer cell line, gracillin markedly suppressed the growth of xenograft tumors derived from patients with breast cancer, suggesting its potential clinical applicability. In addition, major drawbacks of the clinical utilization of metabolism-targeting anticancer agents are side effects and toxicity. In our previous study [32], we have shown that treatment with gracillin displayed minimal effects on the mitochondria function of normal cells derived from human lung epithelium (HBE, 1799, and BEAS-2B), suggesting gracillin might minimally affect the metabolism and viability of normal lung epithelial cells. In addition, we also demonstrated that gracillin did not cause body weight changes, behavioral changes, or functional changes in several organs, including the liver and kidney, indicating gracillin did not cause metabolic disorders, systemic inflammation, or cellular injury [32]. Consistent with these findings, in the current study, the administration of gracillin produced no overt toxic effects with respect to body weight changes. These results suggest the minimal toxicity of gracillin. Additional studies are necessary to confirm the toxicity of gracillin.

The mechanism underlying limited toxicity of gracillin needs to be investigated in further studies, but some possible mechanisms can be suggested as follows. It has been reported that alkylphospholipids, such as perifosine, show detergent-like effects which disrupt the integrity and dynamics of membranes and associated signal transduction, eventually mediating their anticancer, antileishmanial, and leishmanicidal activities [68]. Given the amphiphilic nature of saponins, including gracillin [69], the action mechanism associated with the proapoptotic effect of gracillin may be similar to that of alkylphospholipids. In addition, previous studies have demonstrated that saponins, such as timosaponin, dioscin, and glycyrrhizin, act as a substrate of organic anion-transporting polypeptides, breast cancer resistance protein, and multidrug resistance-associated protein 2 [70]; all have been known to be overexpressed in cancer cells [71,72,73]. Thus, like 3- bromopyruvate, a substrate of monocarboxylate transporter 1 [74], gracillin may utilize these membranous transporters for its cellular uptake and metabolism, which may account for the limited toxicity of gracillin toward normal cells.

Finally, gracillin displays potent inhibitory effects on the viability and colony formation of TNBC cell lines and those carrying acquired resistance to paclitaxel. TNBC is a heterogeneous subtype of breast cancer lacking estrogen receptor (ER), progesterone receptor (PR), and HER2 expression [75,76]. Compared with other types of breast cancer, TNBC is characterized by a distinct molecular profile, aggressiveness phenotypes, such as early onset, large mean tumor size, high grade, early recurrence, aggressive metastasis, and a lack of targeted therapies [76]. Of note, TNBC can utilize both glycolysis and OXPHOS for energy production and has metabolic heterogeneity [77]. Therefore, gracillin may be a potential lead compound to be developed as a single or adjuvant anticancer drug for the treatment of TNBC. Further studies are required to evaluate the antitumor efficacy of gracillin in additional in vitro and in vivo TNBC models. 

## 4. Materials and Methods

### 4.1. Cell Culture

Human breast cancer cell lines (MDA-MB-231 and MCF7) and NSCLC cell lines (H460 and H226B cells) were purchased from the American Type Culture Collection (ATCC; Manassas, VA, USA) or kindly provided by Dr. John V. Heymach (MD Anderson Cancer Center, Houston, TX, USA). T47D cells were kindly provided by Dr. Sang Kook Lee (Seoul National University, Seoul, Korea). MDA-MB-453, BT-20, and MDB-MB-468 cells were kindly provided by Dr. Hyeong-Gon Moon (Seoul National University, Seoul, Korea). The cells were cultured in DMEM (MDA-MB-231, MCF7, MDA-MB-453, and MDA-MB-468 cells) or RPMI 1640 medium (H460, H226B, T47D, and BT-20 cells) supplemented with 10% fetal bovine serum (FBS) and antibiotics (Welgene, Kyeongsan-si, Korea), and maintained at 37 °C with 5% CO_2_ in a humidified atmosphere. Paclitaxel-resistant MDA-MB-231 (MDA/R) cells were generated by continuously exposing the cells to increasing concentrations of paclitaxel for more than 6 months. These cancer cell lines were authenticated and validated using an AmplFLSTR identifier PCR Amplification kit (Applied Biosystems, Foster, CA, USA; cat. no. 4322288) in 2013 and 2017. We used cell lines passed for fewer than three months.

### 4.2. Reagents

A rabbit polyclonal anti-cleaved caspase-3 antibody was purchased from Cell Signaling Technology (Danvers, MA, USA). A mouse monoclonal anti-cleaved PARP antibody was purchased from BD Biosciences (San Jose, CA, USA). Crystal violet, Hoechst 33342, 3-(4,5-dimethylthiazol-2-yl)-2,5-diphenyltetrazolium bromide (MTT), dimethyl sulfoxide (DMSO), and other chemicals were purchased from Sigma–Aldrich (St. Louis, MO, USA) unless otherwise specified.

### 4.3. MTT Assay

Breast cancer cells (2 × 10^3^ cells/well in 96-well plates) were treated with vehicle (100% DMSO) or various concentrations of gracillin for 6 h or 3 days. After incubation, the cells were incubated with an MTT solution (final 500 μg/mL) at 37 °C. After 2–4 h, the culture medium was removed, and DMSO was added to dissolve formazan crystals. The absorbance was detected at 570 nm. The data are expressed as percentages of the vehicle-treated control group. 

### 4.4. Crystal Violet Assay

Cultured breast cancer cells (2 × 10^3^ cells/well in 96-well plates) were treated with vehicle (DMSO) or various concentrations of gracillin diluted in complete medium for 3 days. The cells were fixed with 100% methanol for 30 min at room temperature, air-dried, and then stained with 0.01% crystal violet for 30 min at room temperature. After being washed with tap water multiple times and being dried in air, the stained cells were dissolved in 100% methanol. The absorbance was measured at 570 nm. The data are presented as a percentage of the control group.

### 4.5. Anchorage-Dependent and Anchorage-Independent Colony Formation Assays

For the anchorage-dependent colony formation assay, cells were seeded in 6-well plates at a density of 300 cells/well and then treated with vehicle (DMSO) or increasing concentrations of gracillin diluted in complete medium for 2 weeks. The colonies were fixed with 100% methanol, stained with a 0.01% crystal violet solution at room temperature, and washed with deionized water multiple times. The colonies were counted using ImageJ software (National Institutes of Health, Bethesda, MD, USA) [78].

For the anchorage-independent colony formation assay, before cell seeding, 0.5 mL of 1% low-melting agar solution was poured into 24-well plates and solidified for preparation of a base agar layer. A 0.5 mL cell suspension (0.5–1 × 10^3^ cells/well) mixed with a sterile 1% agar solution (final concentration of 0.4%) was layered onto the base agar. After complete medium with or without gracillin was added to the solidified top agar, the cells were incubated for two weeks at 37 °C with 5% CO_2_. The colonies were stained with an MTT solution (final 500 μg/mL), and then they were imaged and counted using ImageJ software.

### 4.6. Hoechst 33342 Staining

Cells were treated with gracillin (0, 1, and 5 μM) for 2 days and then stained with Hoechst 33342 solution (final 1 μg/mL). Cells were visualized by fluorescence microscopy, and cells with condensed or fragmented nuclei were counted.

### 4.7. Western Blot Analysis

Cells were lysed in a modified RIPA buffer [50 mM Tris-HCl (pH 7.4), 150 mM NaCl, 0.25% sodium deoxycholate, 1% Triton X-100, and 1 mM EDTA] containing various protease and phosphatase inhibitors (100 mM NaF, 5 mM Na_3_VO_4_, 1 mM PMSF, 1 μg/mL aprotinin, 1 μg/mL leupeptin, and 1 μg/mL pepstatin). Equal amounts (30–50 μg) of the cell lysates were resolved using 8% or 15% SDS-PAGE and then transferred to PVDF membranes. The membranes were blocked with blocking buffer [3% BSA in TBS containing 0.1% Tween-20 (TBST)] for 1 h at room temperature and then incubated with primary antibodies diluted in blocking buffer (1:1000) overnight at 4 °C. The membranes were washed with TBST for 1 h at room temperature and then incubated with the corresponding secondary antibodies (GeneTex, Irvine, CA, USA) diluted in 3% skim milk in TBST (1:5000–1:1000) for 1–2 h at room temperature. The membranes were washed several times with TBST for 1 h, and the blots were then visualized using an enhanced chemiluminescence (ECL) detection kit (Thermo Fisher Scientific, Waltham, MA, USA).

### 4.8. Real-Time PCR

Total RNA was isolated using a phenol-chloroform extraction method, reverse-transcribed, and analyzed by PCR. For real-time PCR analysis, an SYBR Green-based qPCR master mix solution was used (Enzynomics, Daejeon, Korea) with gene-specific primers. The primer sequences used for the real-time PCR are as follows: Human *PGK1* forward, GCT GGA CAA GCT GGA CGT TA; human *PGK1* reverse, TGG GAC AGC AGC CTT AAT CC; human *GAPDH* forward, AAC GTG TCA GTG GTG GAC CTG; human *GAPDH* reverse, AGT GGG TGT CGC TGT TGA AGT; and human *ATCB* forward, TCA TTC CAA ATA TGA GAT GCG TTG; human *ATCB* reverse, TAG AGA GAA GTG GGG TGG CT. The thermocycler (ABI Prism 7300, Applied Biosystems) conditions for the real-time PCR analysis were as follows: preincubation at 95 °C for 5 min; 40 cycles of 95 °C for 10 s, 60 °C for 15 s, and 72 °C for 30 s; and melting curve analysis to determine reaction specificity. Relative mRNA expression quantification was performed by the comparative cycle threshold (CT) method, as described previously [79].

### 4.9. Determination of ATP Production

We determined cellular ATP production using ATPlite^TM^ (PerkinElmer, Waltham, MA, USA) according to the manufacturer’s protocol with some modifications. Briefly, cells (8 × 10^3^ cells/well) were seeded in black, clear-bottom 96-well plates (Corning, Corning, NY, USA). After being incubated for 1 day, the cells were treated with vehicle or gracillin (5 μM) for 6 h. After lysis, lysates were treated with the ATPlite substrate. Luminescence was detected on a SpectraMAX M5 multi-reader (Molecular Devices, Sunnyvale, CA, USA).

### 4.10. Determination of Glucose Uptake

Cells were treated with vehicle or gracillin (5 μM) for 6 h. Cells were further stained with 150 μM 2-NBDG (Thermo Fisher Scientific, Waltham, MA, USA) for 30 min. Cells were harvested by trypsinization, and fluorescence was determined by flow cytometry using a FACSCalibur^®^ flow cytometer (BD Biosciences, San Jose, CA, USA).

### 4.11. Fluorescence Imaging Analysis

Cells were seeded in black, clear-bottom 96-well plates (Corning) at a density of 1 × 10^4^ cells/well and treated with vehicle (DMSO) or gracillin (5 μM) for 6 h and then stained with 5 μM MitoSOX (Thermo Fisher Scientific) for 15 min. After counterstaining with Hoechst 33342 (1 μg/mL), fluorescence images were captured and analyzed using the Operetta High Content Imaging System (PerkinElmer).

### 4.12. Measurement of Oxygen Consumption Rate

The basal OCRs in cancer cells were determined by using a Seahorse XF analyzer (Agilent, Santa Clara, CA, USA) according to the manufacturer’s instructions. 

### 4.13. Measurement of Lactate Production

The effect of gracillin on lactate production was determined by using a commercially available kit (BioVision Inc., Milpitas, CA, USA) according to the manufacturer’s instructions. 

### 4.14. Measurement of Extracellular Acidification Rate

The basal ECARs were determined by using a Seahorse XF analyzer (Agilent) according to the instructions provided by the manufacturer.

### 4.15. Enzymatic Activity Assays for GAPDH and PGK

Enzymatic activity assays for GAPDH and PGK were performed using commercially available kits (BioVision Inc., Milpitas, CA, USA) according to the manufacturer’s instructions. To evaluate the effect of gracillin on the activity of these enzymes, cell lysates were preincubated with gracillin (10 μM) for 20 min at room temperature and then treated with the reaction mix according to the manufacturer’s instructions.

### 4.16. LC/MS-Based Metabolite Analysis

H460 and H226B cells were exposed to 5 μM gracillin for 8 h. Metabolites in these cells were analyzed as described previously [80].

### 4.17. Molecular Docking

We performed a molecular docking analysis using the SwissDock (http://www.swissdock.ch) webserver. The PDB codes of GAPDH and PGK1 were obtained from the protein data bank [81]. The image of the binding structure was viewed using JSmol available in SwissDock.

### 4.18. Animal Studies

Animal experiments were performed using protocols approved by the Seoul National University Institutional Animal Care and Use Committee (No. SNU-170228-1). Mice were maintained under standard animal housing conditions (a 12-h light/12-h dark cycle) with free access to standard mouse chow and water. For cell line xenograft experiments, xenograft tumors were generated in the right flank of 6-week-old nonobese diabetic (NOD)/severe combined immune-deficient (SCID) mice by subcutaneous inoculation of MDA-MB-231 cells (2 × 10^6^ cells/mouse). For PDX experiments, breast cancer patient-derived tumors that had been passaged more than 3 times in mice were minced into 2-mm^3^ pieces and subcutaneously inoculated into NOD/SCID mice. After the tumor volume reached 50–150 mm^3^, the mice were randomly grouped and treated with vehicle (1% DMSO and 9% ethanol in distilled water for MDA-MB-231 xenografts; 5% DMSO in corn oil for breast PDX) or gracillin (20 mg/kg) for 2 (MDA-MB-231 xenografts) or 4 (breast PDX) weeks. Tumor growth was determined by measuring the short and long diameters of the tumor with a caliper, and body weight was measured twice per week to monitor toxicity. Tumor volume was calculated using the following formula: tumor volume (mm^3^) = (short diameter)^2^ × (long diameter) × 0.5.

### 4.19. Immunofluorescence Analysis

Sections of formalin-fixed, paraffin-embedded (FFPE) tissue specimens were deparaffinized, rehydrated, and treated with citrate-based antigen unmasking solution (Vector Laboratories, Burlingame, CA, USA) for antigen retrieval. The slides were treated with 0.3% hydrogen peroxide solution and then incubated with blocking solution (5% normal serum in TBS containing 0.025% Triton X-100) for 1 h at room temperature. The slides were incubated with an anti-cleaved caspase-3 antibody (1:100 dilution) at 4 °C overnight. The slides were washed multiple times with wash buffer (TBS containing 0.025% Triton X-100), incubated with a fluorochrome-labeled secondary antibody (Thermo Fisher Scientific) for 1 h, and then washed several times with wash buffer. The slides were counterstained with 4′,6-diamidino-2-phenylindole (DAPI) and observed under a fluorescence microscope. 

### 4.20. In Silico Analysis

We used publicly available datasets (GSE58812 [82], GSE102484 [83], and GSE125989 [84]) deposited in the Gene Expression Omnibus (GEO) database (National Center for Biotechnology Information, Bethesda, MD, USA) to analyze the association of *PGK1* expression with metastasis status in patients with breast cancer. The raw data containing the gene expression levels and clinical information for each patient sample were manually downloaded and analyzed using GraphPad Prism 8 software (GraphPad Software, La Jolla, CA, USA). The normality of the data was determined by using the D’Agostino-Pearson omnibus and the Shapiro–Wilk test. The association of *PGK1* expression with the survival of breast cancer patients was determined by using DRUGSURV (http://www.bioprofiling.de/GEO/DRUGSURV/) [85].

### 4.21. Statistical Analyses

The data are presented as the mean ± SD. All in vitro experiments were performed independently at least twice, and a representative result is shown. Statistical significance was determined by two-tailed Student’s *t*-test, Mann–Whitney test, or Wilkoxon matched-pairs signed-rank test using Microsoft Excel 2010 (Microsoft Corp., Redmond, WA, USA) or GraphPad Prism 8. *p*-values less than 0.05 were considered significant.

## 5. Conclusions

The present study demonstrates the potential of gracillin as an efficacious anticancer agent targeting both glycolysis and OXPHOS. PGK1 is a potential cellular target for the antiglycolytic effect of gracillin. Further studies are warranted to evaluate the effectiveness and toxicity of gracillin in additional preclinical and clinical settings.

## Supplementary Materials

The following are available online at https://www.mdpi.com/2072-6694/12/4/913/s1, Figure S1: Uncropped blots with molecular weight markers and densitometry of each band.
