# Supplementary material for: Potent Anticancer Effect of the Natural Steroidal Saponin Gracillin Is Produced by Inhibiting Glycolysis and Oxidative Phosphorylation-Mediated Bioenergetics
Source: Cancers (Basel). 2020 Apr 8;12(4):913. doi: 10.3390/cancers12040913 (PMC7226187; doi:10.3390/cancers12040913)
Supplement: Supplementary file 1 [file cancers-12-00913-s001.pdf]

Supplementary Materials

Potent Anticancer Effect of the Natural Steroidal Saponin Gracillin Is Produced by Inhibiting Glycolysis and Oxidative Phosphorylation-Mediated Bioenergetics

Hye-Young Min, Honglan Pei, Seung Yeob Hyun, Hye-Jin Boo, Hyun-Ji Jang, Jaebeom Cho, Ji Hye Kim, Jaekyoung Son and Ho-Young Lee

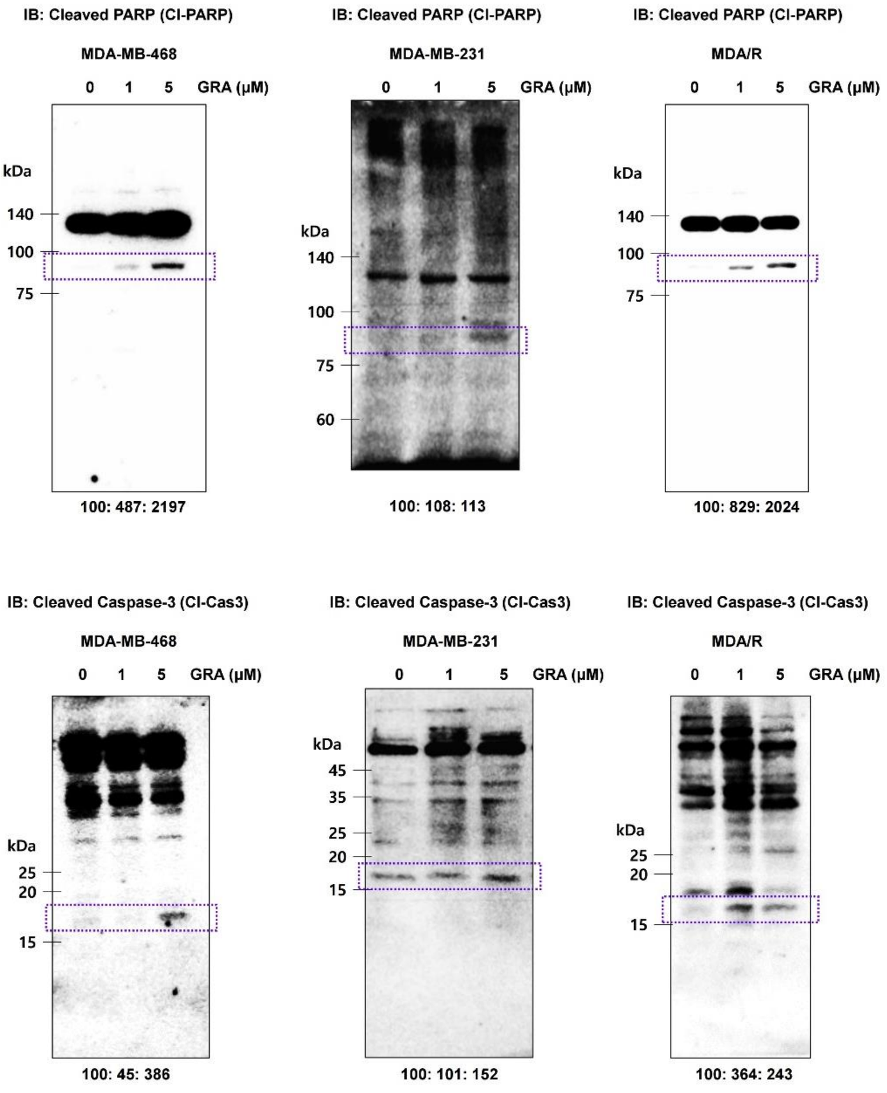

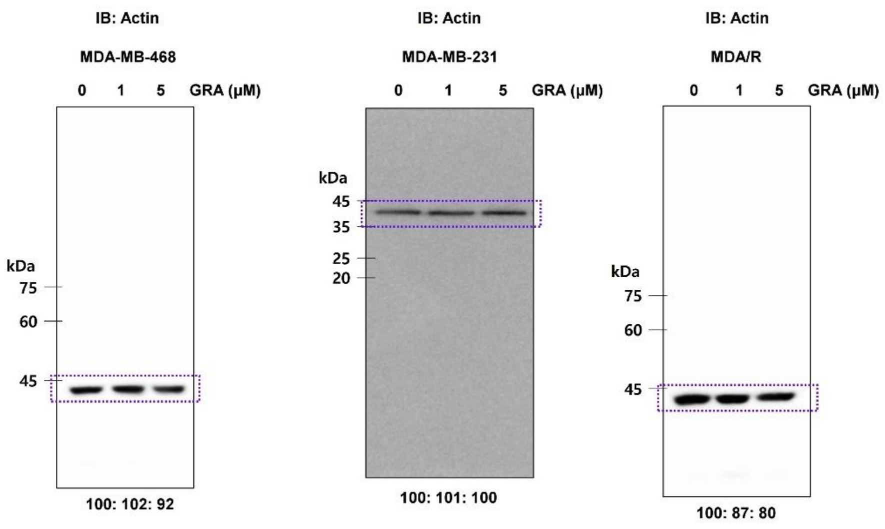

Figure S1. Uncropped blots with molecular weight markers and densitometry of each band.

© 2020 by the authors. Licensee MDPI, Basel, Switzerland. This article is an open access article distributed under the terms and conditions of the Creative Commons Attribution (CC BY) license (http://creativecommons.org/licenses/by/4.0/).
